# AZD8797 is an allosteric non-competitive modulator of the human CX3CR1 receptor

**DOI:** 10.1042/BJ20150520

**Published:** 2016-02-24

**Authors:** Linda Cederblad, Birgitta Rosengren, Erik Ryberg, Nils-Olov Hermansson

**Affiliations:** * Reagents and Assay Development, Discovery Sciences, AstraZeneca R&D Gothenburg, Mölndal 431 83, Sweden; † CVMD Bioscience, Cardiovascular & Metabolic Diseases iMed, AstraZeneca R&D Gothenburg, Mölndal 431 83, Sweden

**Keywords:** allosteric modulator, CX_3_CR1, fractalkine, G-protein, kinetic binding, radioligand binding

## Abstract

The chemokine receptor CX_3_CR1 has been implicated as an attractive therapeutic target in several diseases, including atherosclerosis and diabetes. However, there has been a lack of non-peptide CX_3_CR1 inhibitors to substantiate these findings. A selective small-molecule inhibitor of CX_3_CR1, AZD8797, was recently reported and we present here an in-depth *in vitro* characterization of that molecule. In a flow adhesion assay, AZD8797 antagonized the natural ligand, fractalkine (CX_3_CL1), in both human whole blood (hWB) and in a B-lymphocyte cell line with IC_50_ values of 300 and 6 nM respectively. AZD8797 also prevented G-protein activation in a [^35^S]GTPγS (guanosine 5′-[γ-thio]triphosphate) accumulation assay. In contrast, dynamic mass redistribution (DMR) experiments revealed a weak G_αi_-dependent AZD8797 agonism. Additionally, AZD8797 positively modulated the CX_3_CL1 response at sub-micromolar concentrations in a β-arrestin recruitment assay. In equilibrium saturation binding experiments, AZD8797 reduced the maximal binding of ^125^I-CX_3_CL1 without affecting *K*_d_. Kinetic experiments, determining the *k*_on_ and *k*_off_ of AZD8797, demonstrated that this was not an artefact of irreversible or insurmountable binding, thus a true non-competitive mechanism. Finally we show that both AZD8797 and GTPγS increase the rate with which CX_3_CL1 dissociates from CX_3_CR1 in a similar manner, indicating a connection between AZD8797 and the CX_3_CR1-bound G-protein. Collectively, these data show that AZD8797 is a non-competitive allosteric modulator of CX_3_CL1, binding CX_3_CR1 and effecting G-protein signalling and β-arrestin recruitment in a biased way.

## Abstract (precis)

The present paper shows the non-competitive mechanism by which AZD8797 blocks fractalkine from binding and activating the CX_3_CR1 receptor. CX_3_CR1 is involved in many diseases but, lacking non-peptide ligands, it is poorly investigated. Our work can therefore facilitate drug development.

## INTRODUCTION

CX_3_CL1, also named fractalkine, belongs to the large family of small secreted chemotactic cytokines called chemokines. Their main function is to coordinate leucocyte trafficking in homoeostatic and inflammatory conditions [1]. Chemokines are divided into four sub-families based on the number and arrangement of a conserved cysteine motif (C, CC, CXC and CX_3_C) where CX_3_CL1 is the only known member of the CX_3_C family [2]. CX_3_CL1 is expressed as a membrane-bound molecule with the chemokine domain protruding from a mucin-like stalk, mediating the capture of circulating leucocytes. Additionally, CX_3_CL1 is naturally cleaved, releasing the soluble chemokine domain and, as a consequence, it can act both as a capturing molecule and as a potent chemoattractant [3,4].

**Figure 1:**
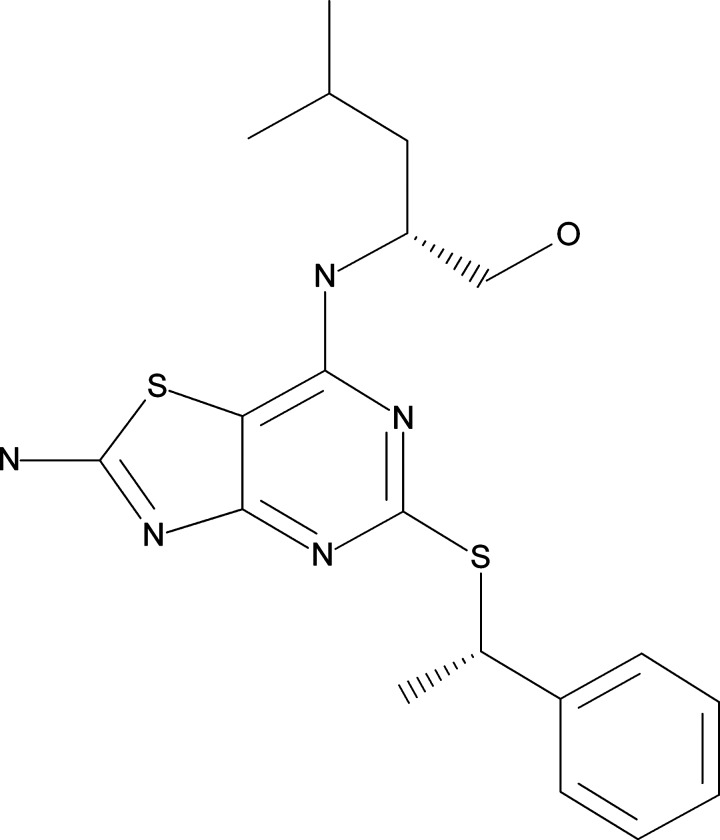
Structure of AZD8797, a low-molecular mass ligand of CX_3_CR1

Unlike most chemokines, CX_3_CL1 interacts with only one G-protein-coupled receptor: CX_3_CR1 [4,5]. CX_3_CR1 is expressed on smooth muscle cells, monocytes, natural killer cells and T-cells and mediates their migration, adhesion and proliferation [6]. Together, CX_3_CL1 and CX_3_CR1 play a key role in the inflammatory responses of several diseases, such as atherosclerosis, diabetes, cancer and pancreatic diseases; for further reading, see [6–8] for reviews. The CX_3_CR1 receptor has consequently been investigated as a therapeutic target. In 2003 a CX_3_CR1 knockout mouse displayed reduced atherosclerosis [9,10]. Using F1, a CX_3_CR1 peptide antagonist, Poupel et al. [11] showed attenuation of atherosclerosis in high-fat-fed APOE (apolipoprotein E) KO mice. CX_3_CR1 antagonism has also been shown to decrease CX_3_CL1-induced extracellular matrix accumulation in diabetic mice [12]. Karlstrom et al. [13] reported the synthesis of the first potent selective and orally available CX_3_CR1 antagonist, 18a. This CX_3_CR1 inhibitor, later named AZD8797 (Figure 1), has since been shown to have efficacy in a rat model for multiple sclerosis [14]. Karlstrom et al. [13] suggested AZD8797 to be an allosteric antagonist, thus affecting the CX_3_CL1 interaction with CX_3_CR1 by binding to the receptor at a different site from that of CX_3_CL1. However, the mechanism of action of AZD8797 has so far not been studied in detail.

Modulation of receptor activation can occur in several different ways. An orthosteric antagonist competes for the same site as the natural agonist. Ligands binding elsewhere on the receptor are referred to as allosteric ligands. These can, both competitively and non-competitively, alter efficacy or affinity of orthosteric ligands. They can also exhibit agonist properties on their own. Furthermore, allosteric ligands can show different, even opposite, effects when looking at different signalling pathways or in different assays on the same pathway [15]. Several allosteric modulators have been found to target chemokine receptors [16–20]. This emerging class of allosteric drugs offers several advantages compared with more traditional competitive antagonists. They often exhibit a higher degree of selectivity and, due to the nature of the allosteric interaction, their effects are saturable, which may provide an increased therapeutic window.

In order to facilitate further *in vivo* and *in vitro* studies on CX_3_CR1, the aim of our study was to extensively characterize the mechanism of action of AZD8797. To this end, we performed functional studies on human whole blood (hWB), B-lymphocyte cells and Chinese hamster ovary (CHO-K1) cells heterologously expressing CX_3_CR1 (CHO-hCX_3_CR1). Using membranes from CHO-hCX_3_CR1 cells, we comprehensively studied the binding properties of AZD8797. We found AZD8797 to be a potent, non-competitive, biased, allosteric modulator of CX_3_CR1.

## MATERIALS AND METHODS

### Materials

Adherent CHO-K1 cells stably expressing hCX_3_CR1 (ES-137-C) were purchased from PerkinElmer. The RPMI-8226 cell line (CCL-155) was purchased from A.T.C.C. The CHO-K1 CX_3_CR1 β-arrestin cell line (93-0290C2) was from DiscoveRx. hWB was collected from healthy volunteers drawn from AstraZeneca staff, giving informed consent to the work. The work was performed in accordance with the Declaration of Helsinki (2013) of the World Medical Association and has been approved by the relevant ethical committee. AZD8797 was synthesized as described previously [13]. AZD8797 is now owned by Acturum Södertälje. [^3^H]AZD8797 (50 Ci·mmol^−1^, 52.932 μM) was labelled in-house, ^125^I-CX_3_CL1 (2200 Ci·mmol^−1^) and [^35^S]GTPγS (guanosine 5′-[γ-thio]triphosphate) (1250 Ci·mmol^−1^) were purchased from PerkinElmer, CX_3_CL1 (8.5 kDa, soluble chemokine domain, unless otherwise stated, was the ligand used) was from Peprotech and recombinant full-length human CX_3_CL1 (365-FR-025/CF) was from R&D Systems. Pertussis toxin (PTX), polyethyleneimine (PEI), GTPγS, GDP and gelatin type A were purchased from Sigma–Aldrich and 3,3′-dihexyloxacarbocyanine iodide (DiOC_6_) was from Molecular Probes. HEPES, Roswell Park Memorial Institute 1640 (RPMI 1640) medium, Ham's F12 (Nutrient mixture F-12 Ham) medium, Dulbecco's modified eagle medium (DMEM), geneticin, phospate-buffered saline (PBS), Hanks' balanced salt solution (HBSS) and Opti-MEM were purchased from Gibco (Life Technologies).

### Cellix flow adhesion

Adhesion of a human B-lymphocyte cell line (RPMI-8226) or hWB leucocytes to full-length human CX_3_CL1 were monitored using a microfluidics Cellix VenaFlux platform and Vena 8 Biochips (Cellix). Each channel on the Vena 8 chip was coated with anti-His monoclonal antibody (R&D Systems) followed by His-tagged full-length CX_3_CL1 (R&D Systems) and blocked for non-specific binding (NSB) with BSA.

Cell preparations: RPMI-8226 cells were cultured in RPMI 1640 medium with GlutaMAX and 20% FBS (HyClone, Perbio). Before the experiment, cells were centrifuged at 320 ***g*** and resuspended to 5 ×10^6^ cells·ml^−1^ in RPMI 1640 with 1% FBS. hWB with heparin as anti-coagulant was collected from healthy donors and 5 μM DiOC_6_ was added. AZD8797 was diluted in a 3-fold series in DMSO starting at 3.3 or 30 μM and pre-incubated with RPMI-8226 cells for 15 min or whole blood for 60 min. DMSO concentration held constant for all AZD8797 concentrations (0.1% for the RPMI-8226 experiments and 0.3% for the whole blood experiments). 100 nM CX_3_CL1 (chemokine domain) was used as the control.

RPMI-8226 cells in RPMI 1640+1% FBS or whole blood were pumped through the channels at 0.5 dyn·cm^−2^ and 2.25 dyn·cm^−2^ respectively, for 3 min. Images for quantification were captured using a microscope. Cells adhering to channels were counted and the mean values across five images in each channel were used for calculating the concentration–response curves.

### CX_3_CR1 membrane preparation

Adherent CHO-K1 cells stably expressing human CX_3_CR1 (CHO-hCX_3_CR1) were grown in F12 medium with GlutaMAX, 10% FBS (Sigma–Aldrich) and 400 μg·ml^−1^ geneticin and harvested at 80% confluency. Cells were washed in PBS and lysed with an Ultra-Turrax Homogenizer in 50 mM Tris/HCl (pH 7.4) and 1 mM EDTA with Complete™ protease inhibitor (Roche). After centrifugation at 1000 ***g*** for 10 min, membranes were collected by ultracentrifugation at 100000 ***g*** for 45 min at 4°C. The membranes were resuspended in 10 mM Tris/HCl (pH 7.4), 1 mM EDTA and 10% sucrose, divided into aliquots, frozen in liquid nitrogen and stored in −80°C. Protein concentration was determined using the BCA method (Thermo Fisher Scientific).

### [^35^S]-GTPγS binding assay

CHO-hCX_3_CR1 membranes (5 μg per well) together with different concentrations of AZD8797 were incubated in 50 mM HEPES (pH 7.4), 100 mM NaCl, 5 mM MgCl_2_, 10 μM GDP and 0.01% gelatin in a MicroWell 96-well plate (Nunc). 0.56 μCi·ml^−1^ [^35^S]GTPγS and EC_80_ of CX_3_CL1 were then added. The plate was incubated at 30°C for 1 h and subsequently unbound [^35^S]GTPγS was separated from bound by vacuum filtration to a Printed Filtermat B (PerkinElmer) using a Skatron Micro96 harvester and dried at 50°C for 1 h. The filters were soaked with a melt-on scintillator sheet (MeltiLex, PerkinElmer), sealed using a MeltiLex heat sealer and measured in a MicroBeta Trilux reader (PerkinElmer). The different AZD8797 concentrations were achieved by stepwise dilution in DMSO to achieve a final DMSO concentration of 1% in all wells after addition of assay buffer, regardless of AZD8797 concentration.

### Dynamic mass redistribution assay

CHO-hCX_3_CR1 cells were seeded at 12000 cells per well in DMEM with 10% FBS and RPMI-8226 cells at 8000 cells per well in RPMI 1640 medium with GlutaMAX and 20% FBS on 384-well fibronectin-coated EPIC™ biosensor plates (Corning) and cultured at 37°C for 24 h. At 18–24 h prior to compound treatment, the medium was replaced with serum-free medium with or without 100 ng·ml^−1^ PTX. Cells were then washed with assay buffer (HBSS, 20 mM HEPES (pH 7.4) and 0.01% BSA) and allowed to equilibrate at 26°C for 1 h inside the EPIC™ plate reader. Following equilibration, a baseline scan was performed before adding AZD8797 or CX_3_CL1 using a CyBi-Well vario (CyBio). Dynamic mass redistribution (DMR) was then measured during a 60 min scan. As in the [^35^S]GTPγS binding assay, the final DMSO concentration was held constant at 1%.

### β-Arrestin assay

β-Arrestin recruitment was measured using the PathHunter™ enzyme fragment complementation assay from DiscoveRx and carried out according to the manufacturer's instructions. In short, 3500 cells per well in Opti-MEM were incubated together with different concentrations of AZD8797 and CX_3_CL1 in a 384-well plate. After 90 min of incubation at 37°C, detection reagent was added and incubation continued for 1 h at room temperature, before reading the plate with a Victor^2^ luminescence plate reader (PerkinElmer). As in the [^35^S]GTPγS binding assay, the final DMSO concentration was held constant at 1%.

### Equilibrium binding studies

For competition binding assays, CHO-hCX_3_CR1 membranes (9 μg per well) together with either 2 nM [^3^H]AZD8797 or 10 pM ^125^I-CX_3_CL1 and different concentrations of competitor were incubated in 25 mM HEPES (pH 7.4), 10 mM MgCl_2_, 1 mM CaCl_2_ and 0.5% BSA in a Corning polystyrene flat-bottom 96-well plate. The plate was incubated for 2 h at room temperature before free radioligand was separated from bound by vacuum filtration on to a Multiscreen HTS+HiFlow FB **(**Millipore) filter plate (pre-soaked with 0.5% PEI for ^125^I-CX_3_CL1 assays), using a Biomek FX (Beckman Coulter). The filter plate was washed in ice-cold 25 mM HEPES (pH 7.4), 5 mM MgCl_2_, 1 mM CaCl_2_ and 500 mM NaCl and dried at 50°C for 1 h. Scintillation cocktail, Optiphase Supermix (PerkinElmer) was added to each well and the radioactivity was measured using a MicroBeta Trilux reader (PerkinElmer).

[^3^H]AZD8797 saturation experiments were performed as above, with radioligand concentrations ranging from 0.5 to 50 nM in the absence or presence of up to 1 μM CX_3_CL1 or 100 μM GTPγS. NSB was measured by adding 10 μM unlabelled AZD8797. For ^125^I-CX_3_CL1 saturation experiments, 1–400 pM radioligand was used in the absence and presence of up to 10 μM AZD8797 or up to 1 mM GTPγS. Unlabelled CX_3_CL1 at 100 nM was used to capture NSB. The DMSO concentration was held constant at 1%.

### Kinetic radioligand-binding studies

Assays were performed as described under equilibrium binding studies with the additions stated below. Association experiments on [^3^H]AZD8797 were performed by adding 1 · *K*_d_ of radioligand in assay buffer in a 96-well plate. The reactions were started by addition of CHO-hCX_3_CR1 membranes at different time points and then incubated for between 1.5 min and 2 h at room temperature before being filtered. For the [^3^H]AZD8797 dissociation experiments 1 · *K*_d_ of radioligand was incubated with CHO-hCX_3_CR1 membranes for 1 h at room temperature before starting the dissociation with the addition of 10 μM unlabelled AZD8797. The reactions were incubated for between 2.5 min and 3 h at room temperature before being filtered.

The association experiments on ^125^I-CX_3_CL1 were performed as above but with 0.25 · *K*_d_ of radioligand, in the absence or presence of up to 100 μM AZD8797 or GTPγS. For the dissociation experiments 0.25·*K*_d_ of ^125^I-CX_3_CL1 was incubated with CHO-hCX_3_CR1 membranes for 2 h at room temperature before CX_3_CL1 and/or AZD8797 and/or GTPγS were added as indicated in the Results section. Incubation times ranged from 2.5 min to 4 h.

### Calculations and data analysis

All analysis and calculations were performed using GraphPad Prism 6.01. Functional and competition binding data were analysed using non-linear regression and fitted to a sigmoidal dose-response with variable slope according to the following equation:

$$Y\;=\;\mathrm{Bottom}+\frac{\mathrm{Top}-\mathrm{Bottom}}{1+10{}^{\wedge }\left(\left(\log\;XC_{50}-X\right)\cdot \mathrm{Hill}\;\mathrm{slope}\right)}$$

where *Y* is the amount of radioligand bound (cpm) or functional effect, Top denotes maximal asymptotic binding, and Bottom denotes the minimal asymptotic binding. *XC*_50_ is either the EC_50_ or the IC_50_ value, and *X* is the logarithm of the test compound concentration. *K_i_* values were calculated from IC_50_ values using the Cheng–Prusoff equation:

$$\;K_{i}=\frac{IC_{50}}{1+\left(\left[\mathrm{radioligand}\right]/K_{d}\right)}\;$$

where *K*_d_ is the radioligand dissociation constant. Saturation binding isotherms were analysed by non-linear regression and globally fitted to a hyperbolic one-site binding model taking into account both NSB and total binding to calculate the total receptor number (*B*_max_) and radioligand dissociation constant (*K*_d_):

$$Y\;=\;\frac{B_{\max}\cdot X}{K_{d}+X}\;+\mathrm{NSB}$$

where *Y* is the total binding expressed in pmol·mg^−1^ CHO-hCX_3_CR1 membranes and *X* is the radioligand concentration. NSB=NS*·X*+Background, where NS is the concentration-dependent NSB.

For the association experiments, the radioligand data were fitted to the following monophasic exponential association equation and the association rate (*k*_on_) was calculated using the added amounts of radioligand (*L*) and dissociation rate (*k*_off_) as constants. *k*_off_ was measured in separate dissociation experiments.

$$Y\;=\;\frac{L\cdot B_{\max}}{L+\left(k_{\mathrm{off}}/k_{\mathrm{on}}\right)}\;\cdot \left(1-e^{-X\cdot \left(k_{\mathrm{on}}\cdot L+k_{\mathrm{off}}\right)}\right)$$

The dissociation data were fitted to the monophasic exponential decay equation:

$$Y\;=\;\left(Y_{0}\;-\mathrm{NS}\right)\cdot e^{-k_{\mathrm{off}}\cdot X)}+\mathrm{NS}$$

where *Y*_0_ is the binding (cpm) at time zero, *X* is the time and NS is the (non-specific) binding at infinite times. Half-life could then be calculated by dividing ln (2) by *k*_off_. Multiple compound dissociation data were fitted to the biphasic exponential decay equation:

$$Y\;=\;Y_{01}\;\cdot e^{-k1_{\mathrm{off}}\cdot X)}\cdot Y_{02}\cdot e^{-k2_{\mathrm{off}}\cdot X)}+\mathrm{NS}$$

*Y*_01_+*Y*_02_+NS equals *Y*_0_ in the monophasic exponential decay equation. *k*1_off_ is the dissociation rate constant for the first phase and *k*2_off_ for the second phase.

All values are expressed as mean or geometric mean (logarithmic data) with 95% CL (confidence limits) unless otherwise stated.

To visualize the manner of binding of CX_3_CL1 and AZD8797, a diagnostic affinity ratio plot designed to compare strictly competitive behaviour with allosteric interactions was used. The plot is that of log (*K*_dapp_*·K*_d_^−1^ − 1) against log [compound], where *K*_dapp_ is the apparent *K*_d_ value measured for the radioligand in the presence of different concentrations of unlabelled test compound. For single site competitive interactions, the resulting linear plot has the slope of 1.0. For allosteric but competitive interactions, the slope will start at 1.0 but progressively deviate from unity, eventually reaching a limit governed by the allosteric ternary complex model co-operativity factor *α* [21,22].

## RESULTS

### Functional studies

**Figure 2:**
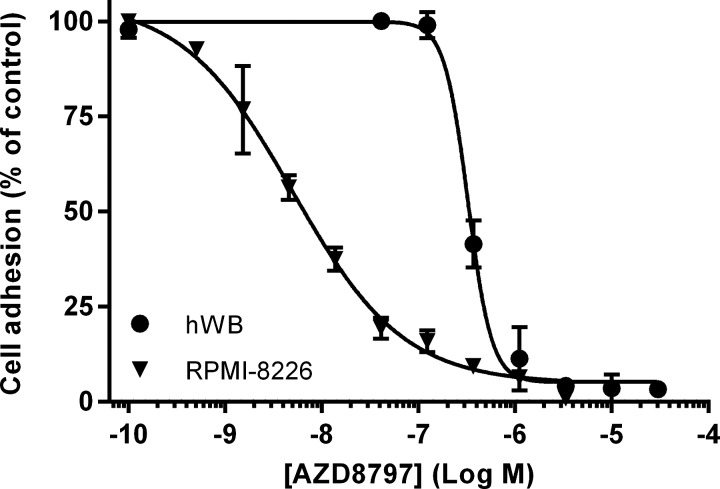
Effect of AZD8797 on adhesion of RPMI-8226 cells or hWB to CX_3_CL1 Each data point is the mean ± S.E.M. for three and two separate experiments respectively. The *y*-axis is normalized to the percentage of adhering cells and compared with untreated control cells.

Circulating leucocytes can adhere to endothelium via interaction between CX_3_CR1 expressed on their surfaces and CX_3_CL1 expressed on endothelial cells. Mimicking physiological flow, we monitored the adhesion of RPMI-8226 cells, a human B-lymphocyte cell line endogenously expressing human CX_3_CR1 and hWB leucocytes to human full-length CX_3_CL1. We found that AZD8797 prevented the capture of RPMI-8226 cells with an IC_50_ of 5.8 nM (4.1–8.2 nM, 95% CL, *n*=3) and human blood leucocytes with an IC_50_ of 330 nM (280–380 nM, 95% CL, *n*=2) (Figure 2). Pre-treatment of cells or blood with soluble CX_3_CL1 produced the same total abolishment of cell adhesion (results not shown).

**Figure 3:**
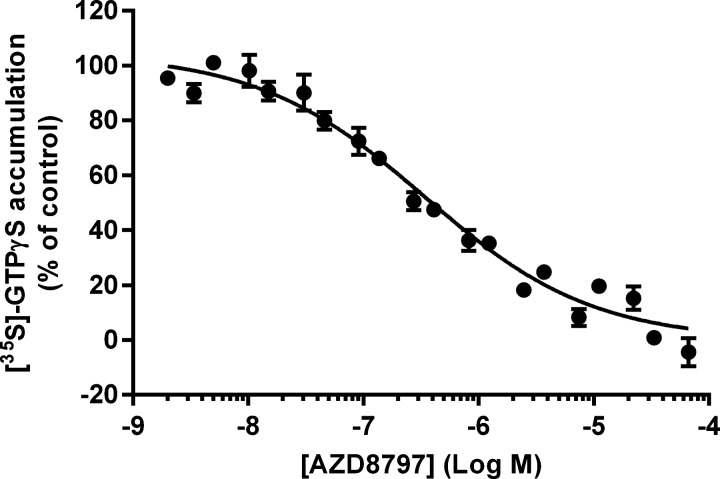
Effect of AZD8797 on CX_3_CL1-induced [^35^S]GTPγS accumulation using CHO-hCX_3_CR1 membranes Each data point is the mean ± S.E.M. for at least 25 separate experiments. The *y*-axis is normalized to the percentage of [^35^S]GTPγS accumulation induced by 2 nM CX_3_CL1.

To study whether AZD8797 could also prevent signalling by CX_3_CR1, a [^35^S]GTPγS accumulation assay was developed. Adding increasing concentrations of AZD8797 to CHO-hCX_3_CR1 membranes fully inhibited the response from 2 nM CX_3_CL1 (EC_80_) with an IC_50_ of 340 nM (250–470 nM, 95% CL, *n* > 24) (Figure 3). No agonistic effect was seen for AZD8797 (results not shown).

**Figure 4:**
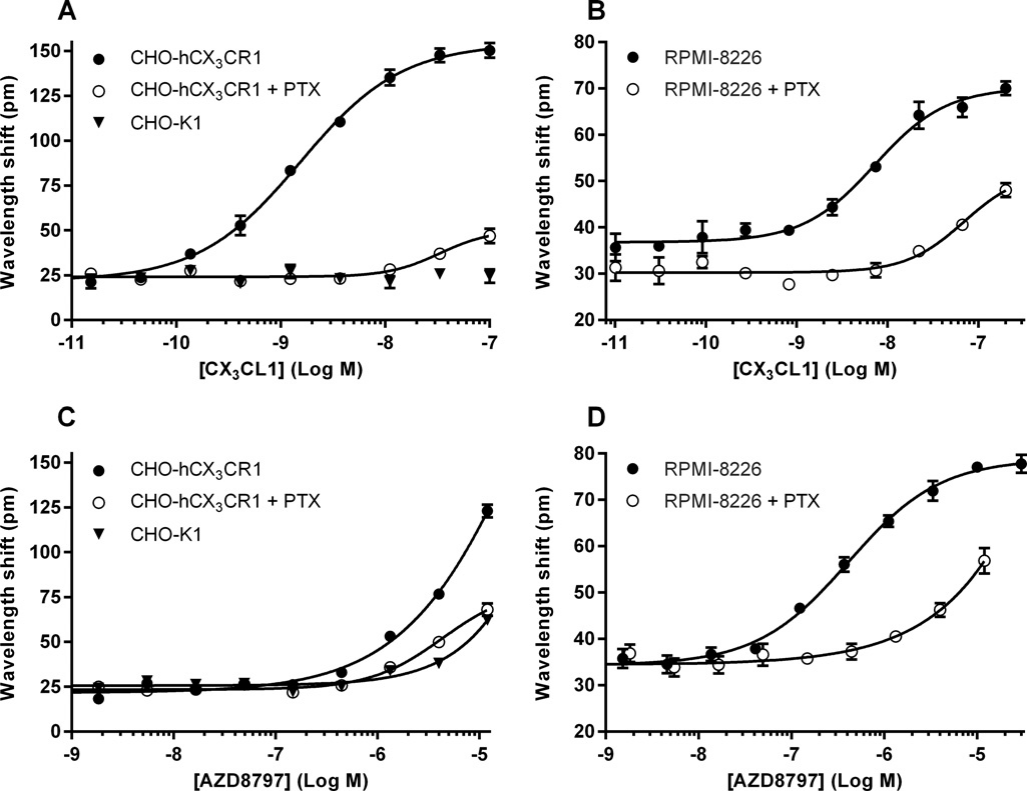
Effect of CX_3_CL1 and AZD8797 on DMR with and without PTX treatment Each data point is the mean ± S.E.M. for three separate experiments. (**A**) Effect of CX_3_CL1 on CHO-K1, CHO-hCX_3_CR1 and PTX-treated CHO-hCX_3_CR1 cells. (**B**) Effect of CX_3_CL1 on PTX-treated and untreated RPMI-8226 cells. (**C**) Effect of AZD8797 on CHO-K1, CHO-hCX_3_CR1 and PTX-treated CHO-hCX_3_CR1 cells. (**D**) Effect of AZD8797 on PTX-treated and untreated RPMI-8226 cells.

To further study the functional response of CX_3_CR1 to AZD8797 we used an EPIC™ reader where we measured the DMR invoked by AZD8797 and CX_3_CL1 treatment in both CHO-hCX_3_CR1 and RPMI-8226 cells. CX_3_CL1 gave an EC_50_ value of 1.5 nM (1.2–1.9 nM, 95% CL, *n*=3) in CHO-hCX_3_CR1 cells (Figure 4A) and an EC_50_ value of 7.3 nM (4.6–12 nM, 95% CL, *n*=3) in RPMI-8226 cells (Figure 4B). We could not measure any antagonist response when challenging EC_80_ of CX_3_CL1 with different concentrations of AZD8797 (results not shown). In both cell lines, however, AZD8797 alone gave rise to signals weak in potency but similar in amplitude to CX_3_CL1. In CHO-hCX_3_CR1, AZD8797 had a low potency of above 5 μM with a maximum effect of 79% of CX_3_CL1 but no plateau (*n*=3) (Figure 4C). RPMI-8226 cells responded stronger to AZD8797 with an EC_50_ value of 410 nM (300–560 nM, 95% CL, *n*=3) (Figure 4D). Both the AZD8797 and the CX_3_CL1 signals were reduced by PTX treatment in both cell types (Figure 4). Additionally, untransfected CHO-K1 cells did not respond to CX_3_CL1 (Figure 4A) and only weakly responded to AZD8797 (38% of AZD8797 effect in transfected cells) (Figure 4C).

**Figure 5:**
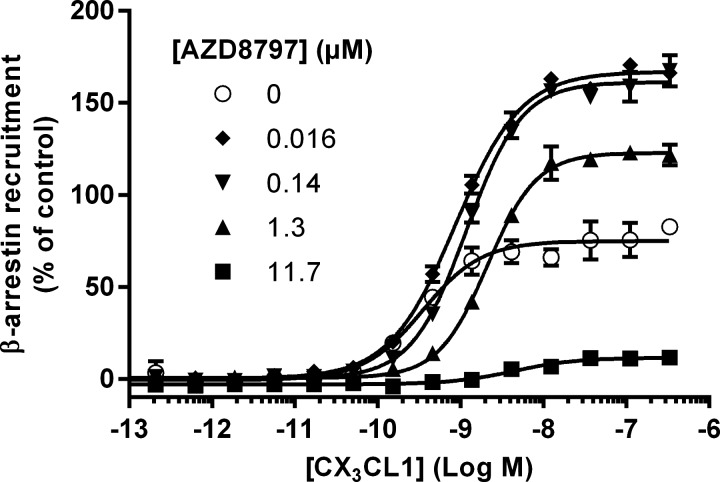
Effect of AZD8797 on β-arrestin recruitment induced by increasing concentrations of CX_3_CL1 Data shown are representative averages and S.E.M. of duplicates from one of three separate experiments. The *y*-axis is normalized to the effect of 1 μM CX_3_CL1 as 100%.

Apart from direct antagonism, β-arrestin recruitment and subsequent internalization of the receptor could also prevent adhesion to CX_3_CL1 and trigger downstream signalling pathways. To elucidate what effect AZD8797 has on the β-arrestin pathway, increasing concentrations of CX_3_CL1 were tested alone and in the presence of different concentrations of AZD8797 (Figure 5). CX_3_CL1 efficacy was increased almost 2-fold using 16 and 140 nM AZD8797, showing a positive allosteric modulation of the CX_3_CL1 response. The effect only enhanced efficacy and not potency. However, at higher concentrations of AZD8797, the CX_3_CL1-induced β-arrestin recruitment was almost abolished. AZD8797 alone showed no effect on β-arrestin recruitment.

### Binding studies

**Figure 6:**
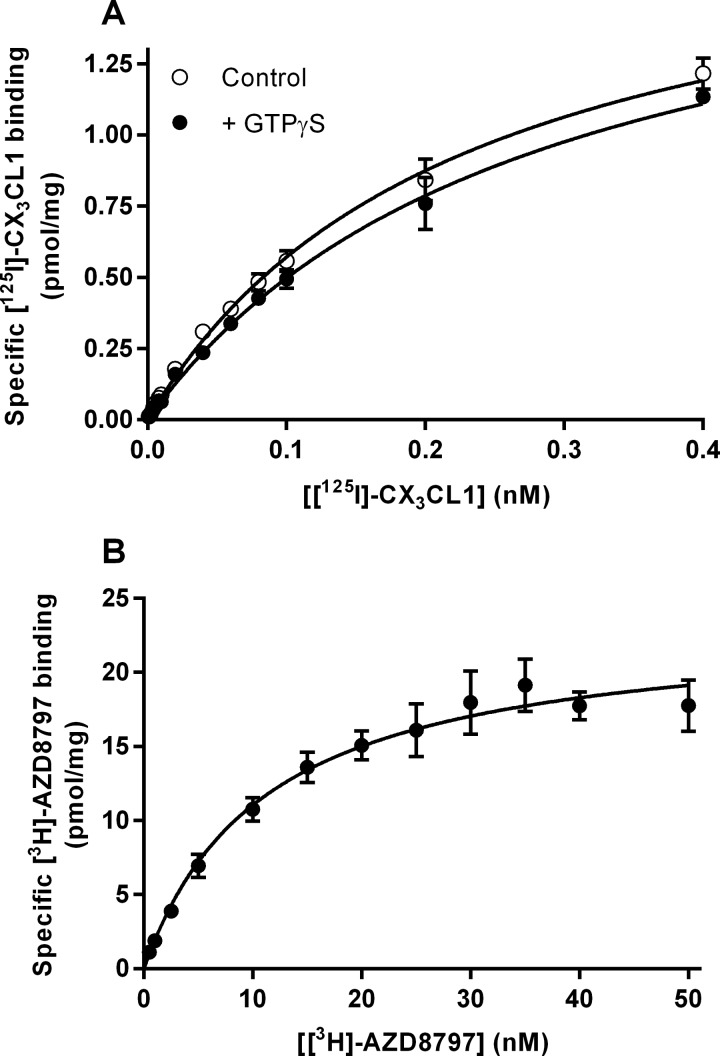
Saturation binding using CHO-hCX_3_CR1 membranes (**A**) ^125^I-CX_3_CL1 saturation binding using CHO-hCX_3_CR1 membranes alone and in the presence of 100 μM GTPγS. Each data point is the mean ± S.E.M. for four and two separate experiments respectively. (**B**) [^3^H]AZD8797 saturation binding using CHO-hCX_3_CR1 membranes. Each data point is the mean ± S.E.M. for four separate experiments.

**Figure 7:**
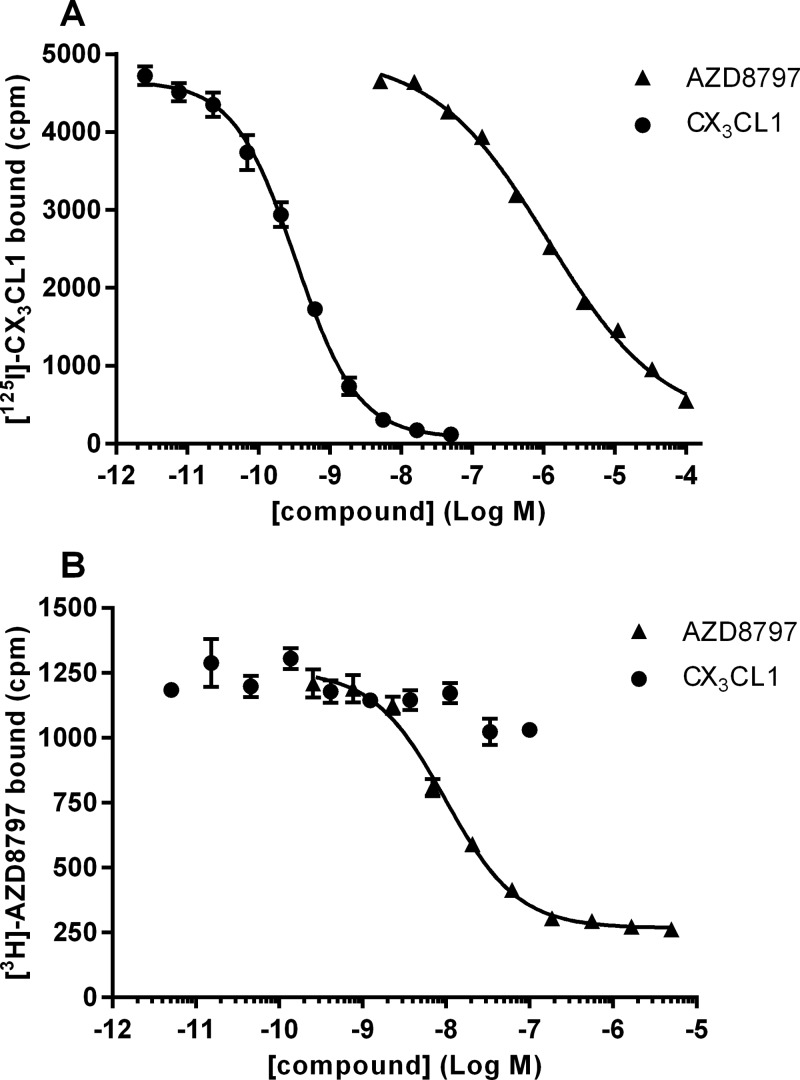
Competition binding using CHO-hCX_3_CR1 membranes (**A**) Effect of CX_3_CL1 and AZD8797 on ^125^I-CX_3_CL1 binding to CHO-hCX_3_CR1 membranes. Data shown are representative averages and S.E.M. of duplicates from one of four and three separate experiments respectively. (**B**) Effect of CX_3_CL1 and AZD8797 on [^3^H]AZD8797 binding to CHO-hCX_3_CR1 membranes. Data shown are representative averages and S.E.M. of duplicates from one of four and seven separate experiments respectively.

To further characterize AZD8797, radioligand-binding studies with [^3^H]AZD8797 and ^125^I-CX_3_CL1 were performed on CHO-hCX_3_CR1 membranes. The binding of ^125^I-CX_3_CL1 was specific and saturable, resulting in a *K*_d_ of 0.19 nM (0.14–0.24 nM, 95% CL, *n*=4) and a *B*_max_ of 1.7 pmol·mg^−1^ (1.3–2.0 pmol·mg^−1^, 95% CL, *n*=4) (Figure 6A). [^3^H]AZD8797 binding was also specific and saturable but with 14-fold more binding sites giving a *B*_max_ of 24 pmol·mg^−1^ (20–28 nM, 95% CL, *n*=4) and a *K*_d_ of 12 nM (11–13 nM, 95% CL, *n*=4) (Figure 6B). Both ^125^I-CX_3_CL1 and [^3^H]AZD8797 saturation data fitted well to the one-site non-linear regression model. Untransfected CHO-K1 cells did not bind [^3^H]AZD8797 in a saturable manner (results not shown). In the ^125^I-CX_3_CL1 competition binding assay, unlabelled CX_3_CL1 competed for CX_3_CR1 binding with a *K_i_* of 0.29 nM (0.20–0.42 nM, 95% CL, *n*=4) (Figure 7A). This fitted well with the determined *K*_d_. In the same assay, AZD8797 was only able to displace ^125^I-CX_3_CL1 with a 1000-fold lower potency giving an EC_50_ of 310 nM (180–520 nM, 95% CL, *n*=3). Unlabelled CX_3_CL1 could not displace or compete with [^3^H]AZD8797 (Figure 7B). Unlabelled AZD8797 competed with [^3^H]AZD8797 for CX_3_CR1 binding with a *K_i_* of 7.3 nM (5.6–9.5 nM, 95% CL, *n*=7), a significantly higher affinity than the potency shown for its displacement of ^125^I-CX_3_CL1. One explanation for the discrepancy in binding sites would be that pre-bound G-proteins stabilize a high agonist affinity subpopulation of CX_3_CR1 receptors. To investigate this, GTPγS was included to uncouple pre-bound G-protein–receptor complexes. In equilibrium saturation binding experiment GTPγS had no effect on neither [^3^H]AZD8797 (results not shown) nor ^125^I-CX_3_CL1 (Figure 6A).

**Figure 8:**
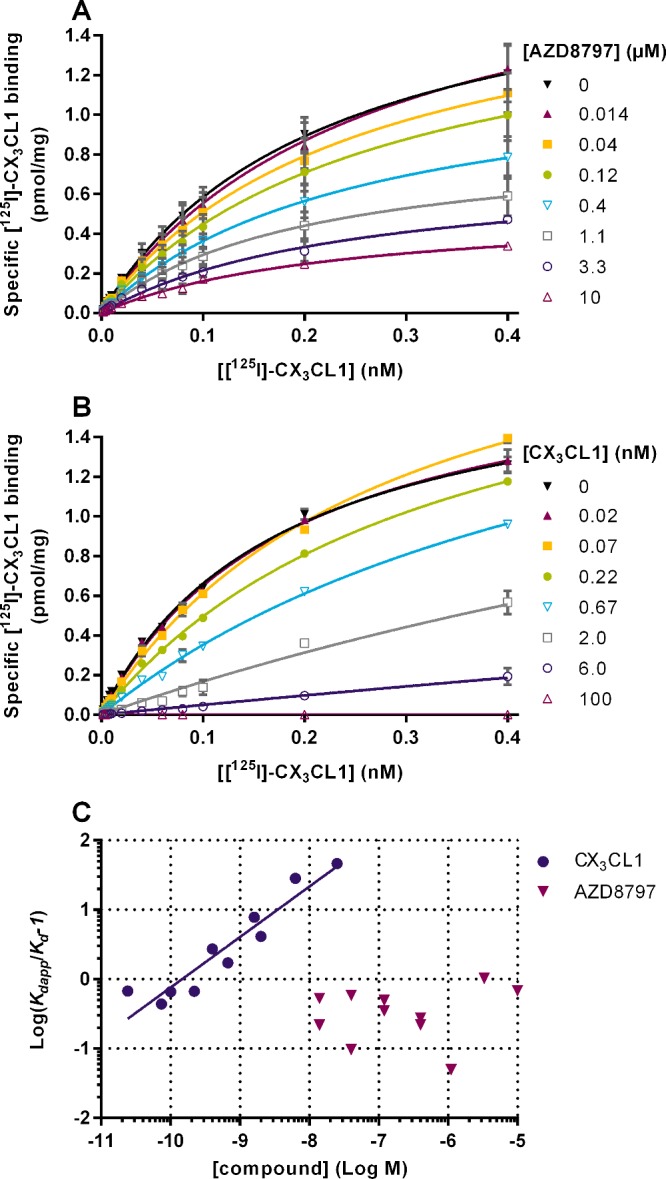
Effect of AZD8797 and CX_3_CL1 on ^125^I-CX_3_CL1 saturation binding to CHO-hCX_3_CR1 membranes (**A**) Specific binding of increasing concentrations of ^125^I-CX_3_CL1 in the presence of different concentrations of AZD8797. Each data point is the mean ± S.E.M. for two separate experiments. (**B**) Specific binding of increasing concentrations of ^125^I-CX_3_CL1 in the presence of different concentrations of CX_3_CL1. Data shown are representative averages and S.E.M. of duplicates from one of two separate experiments. (**C**) An affinity ratio plot of all ^125^I-CX_3_CL1 *K*_d_ values obtained in the presence of either CX_3_CL1 or AZD8797. Data for both CX_3_CL1 and AZD8797 were collected on two separate occasions. CX_3_CL1 linear fit slope 0.73 (0.52–0.93, 95% CL, *n*=2).

To further elucidate the interaction between CX_3_CL1 and AZD8797, [^125^I]-CX_3_CL1 saturation binding was performed in the presence of either AZD8797 or CX_3_CL1. Adding increasing concentrations of AZD8797 decreased the *B*_max_ without any effect on *K*_d_ (Figure 8A). In contrast, unlabelled CX_3_CL1 displayed a competitive behaviour with decreasing *K*_d_ and unchanging *B*_max_ (Figure 8B). Plotting the measured *K*_d_ values in an affinity ratio plot confirmed the competitive nature of the unlabelled CX_3_CL1 towards the ^125^I-CX_3_CL1. These results also clearly demonstrate that AZD8797 is not a competitive allosteric modulator of ^125^I-CX_3_CL1 (Figure 8C).

**Figure 9:**
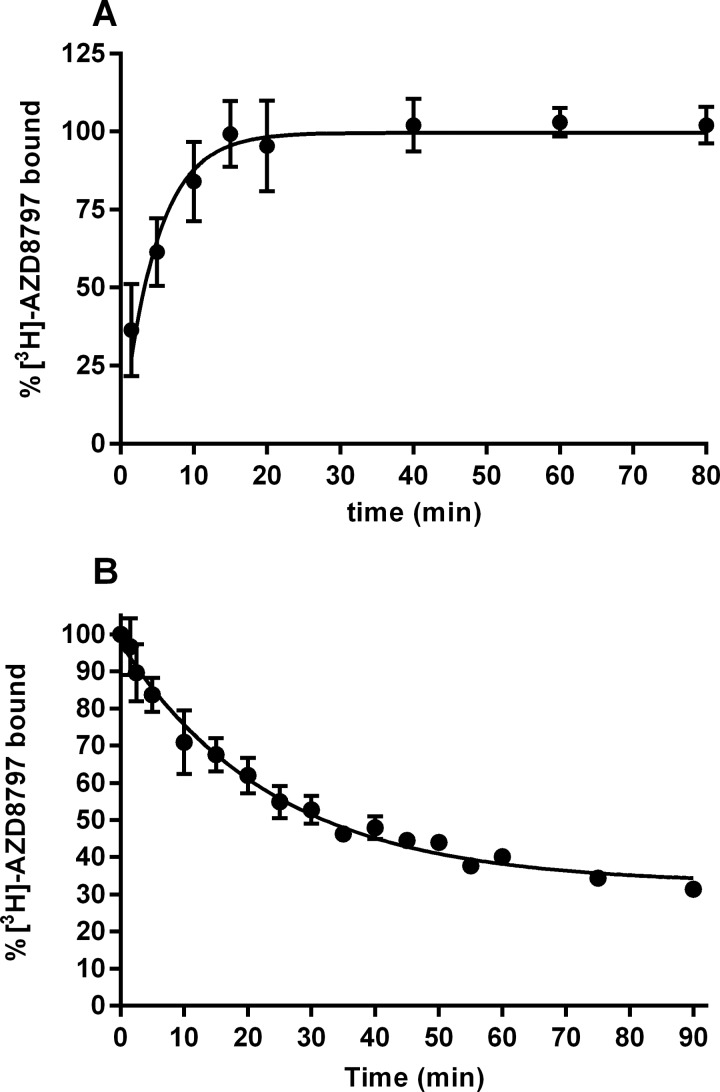
[^3^H]-AZD8797 kinetic binding using CHO-hCX_3_CR1 membranes (**A**) Association of 12 nM [^3^H]AZD8797 to CHO-hCX_3_CR1 membranes. Each data point is the mean ± S.E.M. for five separate experiments. (**B**) Dissociation of 10 nM [^3^H]AZD8797 from CHO-hCX_3_CR1 membranes. Each data point is the mean ± S.E.M. for two separate experiments.

The reduction in binding sites could be the result of AZD8797 displacing CX_3_CL1 in a non-competitive manner or exhibiting very slow dissociation from the receptor. To investigate this, the association and dissociation rates of AZD8797 to hCX_3_CR1 were determined. *k*_on_ was determined to be 1.4×10^7^ M^−1^ min^−1^ (1.1–1.8·10^7^ M^−1^ min^−1^, 95% CL) from the measured *k*_obs_ (*n*=5) (Figure 9A) and *k*_off_ (Figure 9B). *k*_off_ was measured to 0.042 min^−1^ (0.031–0.052 min^−1^, 95% CL, *n*=2), which is equal to a half-life of approximately 17 min. Taken together these rate constants give a *K*_d_ of 2.9 nM, which is fairly close to the *K*_d_ of 12 nM measured in equilibrium binding experiments.

**Figure 10:**
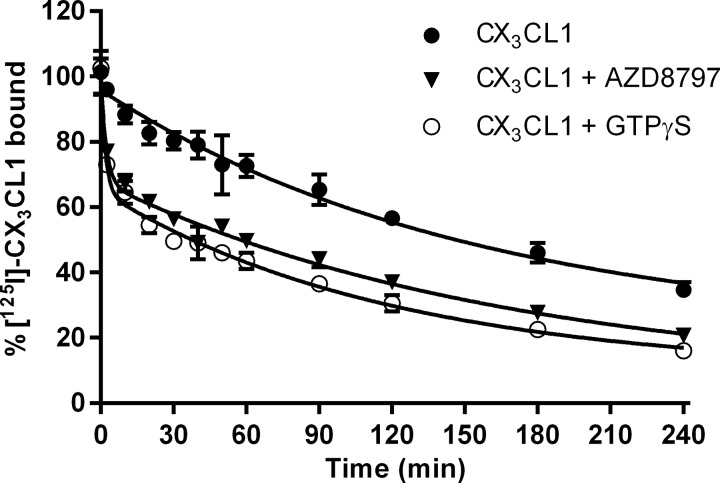
Dissociation of 0.05 nM ^125^I-CX_3_CL1 from CHO-hCX_3_CR1 membranes 100 nM CX_3_CL1 was used to prevent rebinding either alone (*n*=3) or in the presence of 10 μM AZD8797 (*n*=3) or 100 μM GTPγS (*n*=2). Each data point is the mean ± S.E.M. for the indicated number of separate experiments.

Probably the most sensitive method for verifying allosteric interactions is to scrutinize how the proposed allosteric ligand affects the kinetic properties of the orthosteric ligand [23]. Consequently, we measured the association and dissociation of ^125^I-CX_3_CL1 in the absence and presence of AZD8797. Increasing concentrations of AZD8797 decreased maximal binding of ^125^I-CX_3_CL1 in association experiments; however, no effect on *k*_on_ was seen (results not shown). Determining the *k*_off_ revealed a slow dissociation for ^125^I-CX_3_CL1 of 0.0065 min^−1^ (0.0024–0.011 min^−1^, 95% CL, *n*=3), corresponding to a dissociation half-life of approximately 2 h when challenged by a high concentration of unlabelled CX_3_CL1 (Figure 10). An increase in the ^125^I-CX_3_CL1 dissociation rate was observed in the presence of AZD8797 in which the data were best fitted to a two-phase exponential decay model, displaying a fast first phase (*k*1_off_=0.45 min^−1^, 0.18–0.71 min^−1^, 95% CL, *n*=3) followed by a second slower phase (*k*2_off_=0.0060 min^−1^, 0.0014–0.011 min^−1^, 95% CL, *n*=3) (Figure 10).

To assess whether similar effects could be obtained by disrupting receptor–G-protein interactions, GTPγS was included in experiments performed as above. Addition of GTPγS had no effect on the ^125^I-CX_3_CL1 association rate or maximum binding (results not shown). However, when co-adding 100 μM GTPγS a clear increase in the dissociation rate of ^125^I-CX_3_CL1 was observed. Again the data were best described by a two-phase dissociation model (*k*1_off_=0.55 min^−1^, 0.33–0.77 min^−1^, *k*2_off_=0.0084 min^−1^, 0.0049–0.012 min^−1^, 95% CL, *n*=2) (Figure 10). Finally, adding both 100 μM GTPγS and 100 μM AZD8797 as well as 100 nM CX_3_CL1 did not increase the dissociation of ^125^I-CX_3_CL1 further but exhibited the same biphasic pattern as when challenged by 100 nM CX_3_CL1 together with either 100 μM AZD8797 or 100 μM GTPγS (results not shown).

## DISCUSSION

The aim of the present study was to investigate the allosteric mechanism of action of the first selective small-molecule inhibitor published for CX_3_CR1.

In a Cellix flow adhesion assay we studied the interaction between CX_3_CR1 and CX_3_CL1 and showed that RPMI-8226 cells, as well as human blood monocytes, adhere to CX_3_CL1 in a low shear stress environment. This adherence can be totally abolished by addition of soluble CX_3_CL1 or AZD8797. There are, however, a number of ways the cells can be prevented from adhering to CX_3_CL1. To verify that the flow adhesion data were due to AZD8797 functionally antagonizing CX_3_CL1 and thereby preventing downstream signalling of CX_3_CR1, a [^35^S]GTPγS accumulation assay was developed. Using this assay, we observed that AZD8797 indeed prevented CX_3_CL1 from activating the CX_3_CR1-associated G_αi_ protein, albeit with a much lower potency compared with flow adhesion on RPMI-8226 cells. To adhere, a cell needs multiple receptor–CX_3_CL1 interaction points and therefore it is not necessary to remove all CX_3_CL1 interactions for the cell to lose its adherence. This means that the measured potency in the adhesion assay is more a measure of avidity than affinity and cannot be compared directly to the potency achieved in the [^35^S]GTPγS assay.

Using a label-free approach that should be more comparable to the [^35^S]GTPγS assay, AZD8797 surprisingly displayed agonist responses both in RPMI-8226 and in CHO-hCX_3_CR1 cells. The difference in response between CHO-hCX_3_CR1 and untransfected CHO-K1 cells plus the effect of PTX shows the involvement of CX_3_CR1 and G_αi_ in the measured agonist responses, so that they cannot simply be put down to off-target effects. Comparing the effects in CHO-hCX_3_CR1 cells, the concentration of AZD8797 needed to elicit an agonist response is more than 10-fold higher than the concentration needed to block CX_3_CL1 binding and functional response when measured using purified membranes. These results could indicate the activation of other PTX-sensitive pathways not detected by the [^35^S]GTPγS assay, i.e. biased signalling. This can, for example, be achieved via βγ signalling [24]. Regardless of the explanation, these data suggest that AZD8797 is not a classic antagonist.

As the data suggest a receptor conformation upon AZD8797 binding that was not truly inactive and since internalization of the receptor also could present a way to prevent adhesion of CX_3_CR1-expressing cells to CX_3_CL1, we wanted to look at the effect of AZD8797 on β-arrestin recruitment. Additionally, β-arrestin recruitment could trigger downstream signalling pathways. AZD8797 showed a clear positive allosteric modulation of the CX_3_CL1 response at sub-micromolar concentrations. AZD8797 enhanced CX_3_CL1 efficacy, whereas the potency was unchanged. At higher concentrations of AZD8797, the effect was inhibitory. This could indicate that AZD8797 binds allosterically, inducing a conformational change to the receptor that potentiates β-arrestin binding and a potentially functional response while blocking fractalkine binding, albeit with a lower efficiency.

To separate the effects of AZD8797 on CX_3_CL1 efficacy from its effects on CX_3_CL1 affinity, we used radioligand-binding experiments. AZD8797 displaced ^125^I-CX_3_CL1 with similar potency as measured in the [^35^S]GTPγS assay. Allosteric modulation is often reciprocal, i.e. if AZD8797 reduces the binding of CX_3_CL1, the opposite would also occur. However, CX_3_CL1 was unable to compete out [^3^H]AZD8797 binding. This could indicate that the interaction between AZD8797 and CX_3_CL1 is non-competitive, either by AZD8797 being insurmountable due to slow dissociation or by AZD8797 removing binding sites for CX_3_CL1. An interesting finding is that AZD8797 is more than one order of magnitude less potent in displacing ^125^I-CX_3_CL1 than its affinity for the CX_3_CR1 receptor would indicate. Maybe the two most likely explanations for this behaviour are that the ^125^I-CX_3_CL1 and the [^3^H]AZD8797 are assessing different receptor populations or that more than one AZD8797 molecule is needed to displace CX_3_CL1. We also observed that ^125^I-CX_3_CL1 labelled only 7% of the total amount of receptors labelled by [^3^H]AZD8797, further indicating one or the other of the above explanations. However, both Scatchard plots and non-linear comparisons of AZD8797-binding data suggest a one-site model. Finding *B*_max_ differences is not uncommon when comparing agonists with antagonists, where the agonists may only bind to the high-affinity G-protein-bound active conformation, while antagonists bind both the inactive low-affinity and the active high-affinity forms [25,26]. Looking at the influence of guanosine nucleotides in equilibrium binding showed no evidence of a high-affinity form, but, as discussed below, kinetic binding experiments clearly demonstrated its presence.

Next we wanted to verify that AZD8797 works through a non-competitive mechanism. A non-competitive ligand cannot be displaced by increasing concentrations of agonist, i.e. the agonist *B*_max_ should decrease but the agonist *K*_d_ should remain unchanged. Our results confirmed this mechanism of action for AZD8797. As previously discussed, a non-competitive behaviour could be due to a very low compound off rate. We assessed the kinetics of both CX_3_CL1 and AZD8797 and found both to be reversible and surmountable. Therefore, since AZD8797 is able to bind to CX_3_CR1 and non-competitively displace CX_3_CL1, we can surmise that the receptor must be able to simultaneously bind both CX_3_CL1 and AZD8797. The binding of AZD8797, however, decreases the CX_3_CR1 affinity for CX_3_CL1, which therefore should then dissociate from the receptor. Our results demonstrate that AZD8797 indeed profoundly increases the CX_3_CL1 dissociation rate. We could also conclude that a similar effect can be achieved by adding GTPγS, thus removing the G-protein-bound high-affinity receptor forms. Furthermore, no additive effect was seen from GTPγS when co-adding with AZD8797, supporting a mechanism of action for AZD8797 involving G-protein displacement.

Based on the data we have presented we hypothesize that AZD8797 displaces CX_3_CL1 by interfering with the interaction between CX_3_CR1 and the G-protein. The hard to explain G_αi_-dependent agonism seen by DMR could be an effect or an artefact of removing the G-protein from CX_3_CR1. Furthermore, the lowered potency in displacing CX_3_CL1 compared with AZD8797’s own affinity fits well with AZD8797 having a higher affinity for the uncoupled CX_3_CR1 than for the G-protein-bound receptor. Karlstrom et al. [13] reported an almost 100-fold higher affinity of AZD8797 (*K*_i_=4 nM) in a ^125^I-CX_3_CL1 competition assay using human embryonic kidney (HEK293) cells co-transfected with G_qi5_-protein. This could indicate that the G-protein composition is important for the binding of AZD8797. AZD8797 showed a positive allosteric effect in the β-arrestin assay at concentrations comparable to the binding affinity of [^3^H]-AZD8797. However, the antagonist action in the same assay were in the similar range as AZD8797’s ability to displace ^125^I-CX_3_CL1. Studies on the closely related CXCR2 receptor has shown an allosteric mechanism of action of a compound, SB265610, binding intracellularly, close to the site of G-protein coupling and β-arrestin binding [16,27]. Similar binding sites have also been reported for allosteric compounds binding CCR4 and CCR5 [28]. AZD8797 was developed from and is similar to a series of CXCR2 antagonists, with a known intracellular binding site on CXCR2 [29]. The similarities between compounds and receptors together with our data led us to the hypothesis that AZD8797 also binds intracellularly near the C-terminus of CX_3_CR1. Further work using mutagenesis approaches will be required to locate the precise site of interaction between AZD8797 and the CX_3_CR1 receptor.

In conclusion, we have demonstrated that AZD8797 is able to non-competitively displace and block CX_3_CL1 from binding CX_3_CR1 through an allosteric binding mechanism of action. We have also shown that AZD8797 effects G-protein signalling and β-arrestin recruitment in a biased way.

## Abbreviations

CHO-K1: Chinese hamster ovary
CL: confidence limits
DMR: dynamic mass redistribution
GTPγS: guanosine 5′-[γ-thio]triphosphate
hWB: human whole blood
NSB: non-specific binding
PEI: polyethyleneimine
PTX: pertussis toxin

## References

[1] Charo I.F., Ransohoff R.M. (2006). The many roles of chemokines and chemokine receptors in inflammation. N. Engl. J. Med..

[2] Nomenclature, IUIS/WHO Subcommittee on Chemokine (2003). Chemokine/chemokine receptor nomenclature. Cytokine.

[3] Bazan J.F., Bacon K.B., Hardiman G., Wang W., Soo K., Rossi D., Greaves D.R., Zlotnik A., Schall T.J. (1997). A new class of membrane-bound chemokine with a CX3C motif. Nature.

[4] Haskell C.A., Cleary M.D., Charo I.F. (2000). Unique role of the chemokine domain of fractalkine in cell capture kinetics of receptor dissociation correlate with cell adhesion. J. Biol. Chem..

[5] Imai T., Hieshima K., Haskell C., Baba M., Nagira M., Nishimura M., Kakizaki M., Takagi S., Nomiyama H., Schall T.J., Yoshie O. (1997). Identification and molecular characterization of fractalkine receptor CX3CR1, which mediates both leukocyte migration and adhesion. Cell.

[6] White G.E., Greaves D.R. (2012). Fractalkine: a survivor's guide: chemokines as antiapoptotic mediators. Arterioscler. Thromb. Vasc. Biol..

[7] Apostolakis S., Spandidos D. (2013). Chemokines and atherosclerosis: focus on the CX3CL1/CX3CR1 pathway. Acta Pharmacol. Sin..

[8] D'Haese J.G., Friess H., Ceyhan G.O. (2012). Therapeutic potential of the chemokine-receptor duo fractalkine/CX3CR1: an update. Expert Opin. Ther. Targets.

[9] Combadiere C., Potteaux S., Gao J.L., Esposito B., Casanova S., Lee E.J., Debre P., Tedgui A., Murphy P.M., Mallat Z. (2003). Decreased atherosclerotic lesion formation in CX3CR1/apolipoprotein E double knockout mice. Circulation.

[10] Lesnik P., Haskell C.A., Charo I.F. (2003). Decreased atherosclerosis in CX3CR1−/− mice reveals a role for fractalkine in atherogenesis. J. Clin. Invest..

[11] Poupel L., Boissonnas A., Hermand P., Dorgham K., Guyon E., Auvynet C., Charles F.S., Lesnik P., Deterre P., Combadiere C. (2013). Pharmacological inhibition of the chemokine receptor, CX3CR1, reduces atherosclerosis in mice. Arterioscler. Thromb. Vasc. Biol..

[12] Song K.H., Park J., Park J.H., Natarajan R., Ha H. (2013). Fractalkine and its receptor mediate extracellular matrix accumulation in diabetic nephropathy in mice. Diabetologia.

[13] Karlstrom S., Nordvall G., Sohn D., Hettman A., Turek D., Ahlin K., Kers A., Claesson M., Slivo C., Lo-Alfredsson Y. (2013). Substituted 7-amino-5-thio-thiazolo[4,5-d]pyrimidines as potent and selective antagonists of the fractalkine receptor (CX3CR1). J. Med. Chem..

[14] Ridderstad Wollberg A., Ericsson-Dahlstrand A., Jureus A., Ekerot P., Simon S., Nilsson M., Wiklund S.J., Berg A.L., Ferm M., Sunnemark D., Johansson R. (2014). Pharmacological inhibition of the chemokine receptor CX3CR1 attenuates disease in a chronic-relapsing rat model for multiple sclerosis. Proc. Natl. Acad. Sci. U.S.A..

[15] Gregory K.J., Valiant C., Simms J., Sexton P.M., Christopoulos A., Gilchrist A. (2010). The emergence of allosteric modulators for G protein-coupled receptors. GPCR Molecular Pharmacology and Drug Targeting: Shifting Paradigms and New Directions.

[16] Bradley M.E., Bond M.E., Manini J., Brown Z., Charlton S.J. (2009). SB265610 is an allosteric, inverse agonist at the human CXCR2 receptor. Br. J. Pharmacol..

[17] Bertini R., Allegretti M., Bizzarri C., Moriconi A., Locati M., Zampella G., Cervellera M.N., Di Cioccio V., Cesta M.C., Galliera E. (2004). Noncompetitive allosteric inhibitors of the inflammatory chemokine receptors CXCR1 and CXCR2: prevention of reperfusion injury. Proc. Natl. Acad. Sci. U.S.A..

[18] Sabroe I., Peck M.J., Van Keulen B.J., Jorritsma A., Simmons G., Clapham P.R., Williams T.J., Pease J.E. (2000). A small molecule antagonist of chemokine receptors CCR1 and CCR3. potent inhibition of eosinophil function and CCR3-mediated HIV-1 entry. J. Biol. Chem..

[19] Sachpatzidis A., Benton B.K., Manfredi J.P., Wang H., Hamilton A., Dohlman H.G., Lolis E. (2003). Identification of allosteric peptide agonists of CXCR4. J. Biol. Chem..

[20] Dorr P., Westby M., Dobbs S., Griffin P., Irvine B., Macartney M., Mori J., Rickett G., Smith-Burchnell C., Napier C. (2005). Maraviroc (UK-427,857), a potent, orally bioavailable, and selective small-molecule inhibitor of chemokine receptor CCR5 with broad-spectrum anti-human immunodeficiency virus type 1 activity. Antimicrob. Agents Chemother..

[21] Cheng Y., Prusoff W.H. (1973). Relationship between the inhibition constant (K1) and the concentration of inhibitor which causes 50 per cent inhibition (I50) of an enzymatic reaction. Biochem. Pharmacol..

[22] Hulme E.C., Trevethick M.A. (2010). Ligand binding assays at equilibrium: validation and interpretation. Br. J. Pharmacol..

[23] Christopoulos A., Kenakin T. (2002). G protein-coupled receptor allosterism and complexing. Pharmacol. Rev..

[24] Steen A., Larsen O., Thiele S., Rosenkilde M.M. (2014). Biased and g protein-independent signaling of chemokine receptors. Front. Immunol..

[25] Mazzoni M.R., Martini C., Lucacchini A. (1993). Regulation of agonist binding to A2A adenosine receptors: effects of guanine nucleotides (GDP[S] and GTP[S]) and Mg^2+^ ion. Biochim. Biophys. Acta.

[26] Keen M. (1997). Radioligand-binding methods for membrane preparations and intact cells. Methods Mol. Biol..

[27] Salchow K., Bond M.E., Evans S.C., Press N.J., Charlton S.J., Hunt P.A., Bradley M.E. (2010). A common intracellular allosteric binding site for antagonists of the CXCR2 receptor. Br. J. Pharmacol..

[28] Andrews G., Jones C., Wreggett K.A. (2008). An intracellular allosteric site for a specific class of antagonists of the CC chemokine G protein-coupled receptors CCR4 and CCR5. Mol. Pharmacol..

[29] Nicholls D.J., Tomkinson N.P., Wiley K.E., Brammall A., Bowers L., Grahames C., Gaw A., Meghani P., Shelton P., Wright T.J., Mallinder P.R. (2008). Identification of a putative intracellular allosteric antagonist binding-site in the CXC chemokine receptors 1 and 2. Mol. Pharmacol..

